# Chronic Benign CD8+ Proliferation: A Rare Affection that Can Mimic a Lymphoma Relapse

**DOI:** 10.1155/2019/4932616

**Published:** 2019-03-05

**Authors:** Marcela Osovská, Andrea Janíková, Leoš Křen, Andrea Marečková

**Affiliations:** ^1^Department of Hematology and Oncology, University Hospital Brno, Faculty of Medicine, Masaryk University Brno, Brno, Czech Republic; ^2^Department of Pathology, University Hospital Brno, Faculty of Medicine, Masaryk University Brno, Brno, Czech Republic; ^3^Department of Molecular Biology and Genetics, University Hospital Brno, Faculty of Medicine, Masaryk University Brno, Brno, Czech Republic

## Abstract

Chronic benign CD8+ proliferation is a rare syndrome that can take the form of a variety of other diseases. Peripheral adenopathy, cytopenia, and infiltration of the liver, kidneys, bowels, or other organs are the most common clinical presentations of the syndrome. CD8+ expansion can be clonal and nonclonal. It generally occurs in patients with innate or acquired immunodeficiency (HIV+) or in patients receiving immunosuppressive therapy. It has been found repeatedly in patients who developed severe hypogammaglobulinemia after treatment with rituximab. Diagnosis of the disease can be difficult because it can mimic relapse of a lymphoma, and a common biopsy examination cannot identify the problem at first. The authors describe a case of a patient pretreated with rituximab who developed agammaglobulinemia and peripheral adenopathy. Biopsy of an enlarged lymph node showed “reactive lymphadenitis.” Additionally, a flow-cytometric examination revealed a pathological population of CD8+ lymphocytes. The treatment, which differed from treatments of lymphoma relapse, consisted of corticosteroids and IVIG substitutions and has led to a regression of clinical symptoms. With more frequent usage of rituximab, one can expect increased occurrence of a very rare CD8+ expansion that can reliably emulate the relapse of a lymphoma.

## 1. Introduction

Chronic CD8+ T-cell proliferation is a rare heterogeneous group of disorders which is characterized by an increased population of CD8+CD3+ cells in the tissues and organs, morphologically in the form of large granular lymphocytes. The expansion of CD8+ lymphocytes comprises a population of activated cytotoxic T-cells in the final phase of their development and can be of clonal (reactive) or nonclonal origin [1, 2]. The CD8+ expansion was previously documented in conditions connected to immunodeficiency (e.g., in patients with common variable immunodeficiency, in HIV-positive patients, and in patients undergoing immunosuppressive therapy).

CD8+ lymphoproliferation in HIV patients is thought to be a part of the immune system's reaction to HIV (although it is not present in all such patients) and can take two forms: (1) CD8+ lymphocytosis and (2) diffuse infiltrative lymphocytosis syndrome (DILS). DILS is typical of sequestration of CD8+ cells into the salivary glands, lymph nodes, lungs, kidneys, and other organs, displaying a clinical similarity to Sjögren's syndrome [3–6]. Furthermore, in patients with CVID, a cytotoxic T-lymphoproliferation can be sometimes associated with liver impairment reminiscent of a lymphoma (nodular regenerative liver hyperplasia) [7, 8].

Over the past two decades, rituximab has shown good effectiveness in the treatment of B-lymphomas, both as a single agent and in combination with conventional chemotherapy [9–11]. Rituximab is a chimerical monoclonal anti-CD20 antibody, of which the mechanism of the effect in humans is not fully known. The CD20 antigen is expressed on the surface of B-lymphatic cells (excluding lymphoblasts, pre-B-cells, and plasmacytes), both benign and malignant. In nonmalignant cells, the linkage to the CD20 antigen leads to the inhibition of cell activity, proliferation, differentiation, and lowering of immunoglobulin secretion. Compared with chemotherapy, rituximab appears to be practically nontoxic. However, the anti-CD20 antibody has affected the immune system of several patients. In certain cases, especially in repeated doses, it causes clinically apparent immunodeficiency. Infections and neutropenia, usually combined with severe hypogammaglobulinemia, are the most common long-term side effects [12–14].

In the following text, we present a case of the still rather rare chronic CD8+ lymphoproliferation in a patient with follicular lymphoma who had been treated repeatedly with rituximab. Only a few patients worldwide have been reported with a similar diagnosis. The authors assume that, with long-term and massive repeated use of the anti-CD20 antibodies for B-lymphoproliferation treatments, the number of patients with this secondary affection will increase. The diagnosis of the disease can be difficult because it can easily evoke a relapse of the former lymphoma and the development of CD8-lymphoproliferation can occur with a significant delay after the end of immunochemotherapy.

## 2. Case Report

A 78-year-old female with follicular lymphoma came to the hematology-oncology department for a routine checkup in May 2016. She had been suffering from intermittent fevers, tiredness, significant weight loss, and night sweats for several weeks. She was treated repeatedly with antibiotics with no clinical effect. Up to that point, the patient was still working and was an active woman with no other diseases or chronic medication.

Initially, the patient was diagnosed with a follicular lymphoma (FL) of stage IIIA in 1999 and underwent standard treatment consisting of 6 cycles of CHOP with the achievement of a complete remission. In 2003, the patient developed asymptomatic, low-burden, histology-proven relapse of indolent FL, but therapy was only started in October 2004, when bulky disease and symptoms were revealed. The patient was treated with 6 cycles of R-COPP immunochemotherapy, resulting in a complete remission.

Since 2015, PET/CT had shown mild lymphadenopathy (up to 25 × 15 mm; SUVmax up to 5.79), but the patient remained asymptomatic and no treatment was administered. In May 2016, the progression of cervical lymphadenopathy and systemic symptoms occurred, accompanied by a significant elevation of the liver enzymes, LDH, and CRP (ALT 6.01 *μ*kat/l, AST 5.96 *μ*kat/l, ALP 22.69 *μ*kat/l, GGT 20.40 *μ*kat/l, LDH 8.19 *μ*kat/l, and CRP 27.6 mg/l). However, lymph biopsy showed no evidence of malignant cells; only reactive inflammatory infiltration with central coliquation was found. The patient's condition was deteriorating rapidly with continuous fevers, and she developed ascites. Serological and PCR tests (CMV, EBV, hepatitis A, B, C, HIV, HHV-8, chlamydia, and mycoplasma) gave negative results. The bone marrow and paracentesis were free of pathology. Flow cytometry identified an elevated count of T-lymphocytes and a nearly zero level of B-lymphocytes concordant with deep hypogammaglobulinemia (IgG < 0.4 g/l, IgA < 0.05 g/l, and IgM = 0.09 g/l). Thorough immunological analysis revealed a significant elevation of CD8+ cells in the blood up to 57% (normal 39%), mildly elevated CD3+ cells; conversely, the population of CD19+ was almost undetectable. The examination of the liver, portal system, and biliary tract by CT and ultrasound showed no pathology.

In conclusion, based on a finding of severe B-lymphocytopenia, agammaglobulinemia, and unusual CD8+ cell population, we assumed that our patient suffered from a rare CD8+ lymphoproliferation. We performed additional special (not routinely performed) staining of bone marrow and lymph node specimens targeted at the CD8+ cells, where an evident clonal CD8+ cell population was found. Corresponding T-cell clonality in the peripheral blood and bone marrow was also confirmed.

There is currently no standard treatment available for this disorder. Based on published case reports, we administered a combination of intravenous immunoglobulin (IVIG 0.3 g/kg—once every three weeks) and corticosteroids (initial dosage of prednisone 1 mg/kg/day) [1]. Within four weeks, the patient's condition had improved significantly, lymphadenopathy diminished, ascites and systemic symptoms disappeared, and liver enzymes decreased (ALT 1.71 *μ*kat/l, AST 1.32 *μ*kat/l, ALP 7.32 *μ*kat/l, and GGT 11.41 *μ*kat/l). Thus, we could slowly reduce the dosage of corticosteroids. Since April 2017, the patient has been asymptomatic with laboratory tests within the norm and now is on a small dose of prednisone (5 mg/day). Up to now, the patient is well without symptoms of lymphoma or CD8+ lymphoproliferation, but regular IVIG substitution is necessary.

## 3. Discussion

So far, only a few cases of chronic CD8+ T-lymphoproliferation have been described. One of the largest published studies, with 14 patients, reported only two cases of CD8+ proliferation after rituximab treatment. One patient received rituximab maintenance, and another was treated with rituximab plus a fludarabine-based regimen. At the time of the CD8+ lymphoproliferation diagnosis, both patients (similarly to our patient) were in complete remission and had developed significant hypogammaglobulinemia. The first patient developed CD8+ expansion one year after the rituximab maintenance; it was manifested by aseptic CD8+ lymphocytic meningitis with fever, diarrhea, and lymphocytosis, and with bone marrow CD8 lymphocyte infiltration. The patient was treated successfully with intravenous immunoglobulins (0.5 g/kg every 3 weeks for 6 months), and rituximab was discontinued. After this treatment, the neurological symptoms resolved completely and the CD8+ T-cell lymphocytes disappeared from the CSF. The second patient developed unusually severe pancytopenia during rituximab-containing therapy, associated with persistent diarrhea and portal hypertension with ascites because of regenerative nodular hyperplasia. The CD8+ T-cell lymphocytes were present in the ascites liquid, in the peripheral blood, and in the bone marrow. A colon biopsy showed mucosal CD8+ T-cell infiltration. The patient's condition improved dramatically, and the blood cell count recovered after intravenous immunoglobulin treatment (0.5 g/kg every 3 weeks for 6 months) [1].

Another case report discusses a patient with long-term hypogammaglobulinemia persisting for 6 years after the end of rituximab-based therapy. The patient developed severe hypogammaglobulinemia within 10 months after end of the treatment with zero levels of IgG, IgA, and IgM, and no clinical symptoms. Before the treatment, the patient's globulin level was within the normal limits, the level of IgG dropped below 1 g/l, and IgA and IgM became undetectable. The patient was HIV-negative, with no family history of immunodeficiency, and had maintained complete remission since 2009. The levels of immunoglobulins have shown no changes after intravenous treatment (dose 5 g/month) for 6 years despite the recovery of the peripheral B-cell counts [15].

In the mentioned cases, hypogammaglobulinemia developed within a maximum of 1 year after the rituximab treatment. However, our patient developed immunodeficiency 11 years after the end of the treatment. Therefore, it is necessary to consider the diagnosis of CD8+ lymphoproliferation as a possible diagnosis even in patients with long-term remission of lymphoma. A biopsy with focused staining for CD8+ should be recommended as part of the histological algorithm, especially in cases of nonspecific “inflammatory” changes in the sample. With the massive use of CD20 antibody, we expect to see more cases with CD8+ expansion in such patients in the future. It is difficult to identify CD8+ lymphoproliferation during a standard histology examination of the tissue.

According to several studies, rituximab combined with chemotherapy leads to a loss of cell differentiation, increase in B-cell apoptosis, and changes in the T-cell population, which results in panhypogammaglobulinemia. The mechanism of invocation of the CD8+ lymphoproliferation after rituximab is caused by the CD27+IgM-IgD memory cell reduction. The B-cells stop their differentiation into the memory cells or plasmocyte and they produce less IgG. There are also secondary changes in the T-cell population. The expanded CD8+ T-lymphocytes represent activated T-lymphocytes at a terminal stage of their differentiation, which have usually lost their cytotoxic properties to become effector memory T-lymphocytes. The CD8+ T-cell expansions can result in symptomatic organ infiltration including the blood and bone marrow. They can also manifest in a variety of symptoms such as cytopenia, autoimmune disease, hepatitis, colitis, or lymphadenitis.

## 4. Conclusion

Although chronic benign CD8+ proliferation is a rare syndrome, we have to consider this diagnosis with immunocompromised patients and patients after rituximab treatment. It can mimic a systemic relapse of lymphoma with its typical laboratory and clinical symptoms, but a misdiagnosis with the administration of chemotherapy in those cases may be fatal. The diagnosis of the disease can be difficult, and it is necessary to know about this possibility. A standard biopsy examination has to be performed, but it will not identify the problem at first, which is a problem of which we have to be aware.

## Abbreviations

CNS: Central nervous system
CSF: Cerebral spinal fluid
CMV: Cytomegalovirus
CVID: Common variable immunodeficiency
DILS: Diffuse infiltrative lymphocytosis syndrome
EBV: Epstein–Barr virus
HAV: Hepatitis A virus
HHV-8: Human herpes virus
IVIG: Intravenous immunoglobulin
PET/CT: Positron emission tomography and computed tomography
R-COPP: Rituximab, cyclophosphamide, vincristine, procarbazine, and prednisone
R-CHOP: Rituximab, cyclophosphamide, vincristine, doxorubicin, and prednisone.
